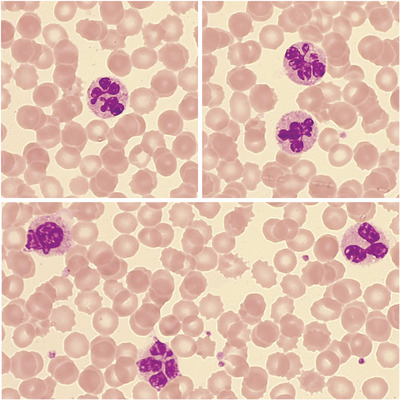# Botryoid neutrophils in sepsis caused by influenzae virus A

**DOI:** 10.1002/jha2.82

**Published:** 2020-09-18

**Authors:** Maite Serrando, Anna Marull, Orlando Jiménez

**Affiliations:** ^1^ Hematology Laboratory Institut Catala De La Salut Girona Spain; ^2^ Haematology Laboratory Hospital Universitari de Girona Doctor Josep Trueta Girona Spain

**Keywords:** cytology, cytomorphology, sepsis

## Abstract

A 13‐year‐old patient was admitted to our hospital with severe respiratory distress, fever, and signs of meningeal infection. A positive result of swine flu was obtained by RT‐PCR. All bacterial cultures were negative. Due to a worsening progression, the patient required mechanical ventilation. Leucocytosis with neutrophilia was observed with an absence of immature granulocytes and thrombocytopenia. A blood film was therefore examined, observing 85% of botryoid neutrophils, hypersegmented, and radially distributed nuclear lobs, and a large number of apoptotic cells. There was an elevated nucleus‐cytoplasm ratio and neutrophils presented as slightly degranulated. The detection of botryoid nuclei in the neutrophils is a very infrequent finding and are usually found in cases of severe burns, hyperthermia, abuse of cocaine and methamphetamine, and encephalitis. Current studies also suggest that some morphometric measurements (cell population data [CPD]) can be altered in the case of sepsis and serious infection. In this particular case, [NE‐SSC] = 159.9, [NE‐FSC] = 94.6, and [NE‐SFL] = 65.5, which correspond to measures of cellular complexity, size, and fluorescence and were above normal threshold values (performed by Sysmex XN20). Current studies directly relate the increase of NE‐SSC > 149 in patients presenting with sepsis. The CPD and abnormal neutrophil morphology improved as the patient recovered. Results normalized by day 30 postdischarge.

## 1 INTRODUCTION

The Third International Consensus defines sepsis as a life‐threatening organ dysfunction caused by a dysregulated host response to infection. Sepsis produces an innate immune response leading to morphological changes in cells that can alter their size and complexity. These changes can be visualized in the full blood count (CBC) both quantitatively (number of leukocytes, immature granulocytes, monocytes, and/or lymphocytes) and qualitatively (using analyzer flags). Current studies also suggest that some morphometric measurements (such as cell population data [CPD]) can be altered in the case of sepsis and serious infection.

At the cytological level, we can observe relevant morphological alterations. The detection of botryoid nuclei in the neutrophils is a very infrequent finding. These cells present an abnormal distribution of the chromatin: hypersegmented nuclei, radial pattern arrangement, and interchromatin bridges. Botryoid nuclei are usually found in cases of severe burns, hyperthermia, abuse of cocaine and methamphetamine, and encephalitis. We present a clinical case of a 13‐year‐old patient diagnosed with encephalitis due to influenza virus A (swine flu).

## 2 CLINICAL CASE

A 13‐year‐old patient was admitted to our hospital with severe respiratory distress, fever (39,1ªC), and signs of meningeal infection. A positive result of swine flu was obtained by RT‐PCR. All bacterial cultures were negative. She was treated empirically with broad‐spectrum antibiotics. Due to a worsening progression of symptoms, the patient required mechanical ventilation and was admitted to the ICU.

The CBC reveals moderate leucocytosis with neutrophilia, an absence of immature granulocytes, and thrombocytopenia. Cell population data (Sysmex XN20) yielded the following results: [NE‐SSC] = 159.9, [NE‐FSC] = 94.6, and [NE‐SFL] = 65.5, which correspond to measures of cellular complexity, size, and fluorescence and were above normal threshold values. In the peripheral blood smear, we observed neutrophils with mature, hypersegmented, and radially distributed nuclear lobs. There was also an elevated nucleus‐cytoplasm (NC) ratio and neutrophils presented as slightly degranulated. We observed 85% of neutrophils containing botryoid nuclei and a high number of apoptotic cells. The CPD as well as the presence of abnormal neutrophils morphology improved as the patient recovered. Results normalized by day 30 postdischarge.

## 3 DISCUSSION

Morphometric values are of great use in the diagnosis of sepsis. Current studies directly relate the increase of NE‐SSC > 149 in patients presenting with sepsis. In this case study, the elevated neutrophils count and CPD relate also to cellular morphology.

Severe cases of swine flu infection have been associated with the development of neutrophil extracellular traps (NETs), which trigger inflammation, aggravated by the immune system and delayed apoptosis.

This inflammatory response does not appear likely to be related to abnormalities such as leucocytosis; however, the morphological changes produced by a neutrophil‐mediated inflammatory response can affect their phenotypic expression. These alterations are reversible as shown in the clinical case we present. Results indicated that all lab values returned to normal after the acute episode.